# Answer to the letter by Frass et al. to our systematic review

**DOI:** 10.1007/s00432-022-04239-z

**Published:** 2022-08-09

**Authors:** J. Hübner, Ch. Lübbers

**Affiliations:** 1 Klinik Für Innere Medizin II, Universitätsklinikum Jena, Jena, Germany; 2 Informationsnetzwerk Homöopathie, Roßdorf, Germany

We appreciate the letter by Frass et al. to our systematic review with focus on our risk of bias assessment of two of the studies included. It shows that prominent researchers in homeopathy acknowledge our results and apparently share our concerns about these two studies we detailed in our comments section.

In detail, the authors of the letter argue on two manuscripts by Frass et al. (2015 and 2020). In fact, both studies have severe drawbacks that led to our RoB ratings that we did not fully describe due to word count limitations. But we would like to get into more detail here.

## Frass et al. (2020)

We have reviewed the paper together with the information available on clinicaltrials.gov (https://clinicaltrials.gov/ct2/show/NCT01509612) including the history of changes. We have some serious concerns that the results are a product of strong biases arising from modifications of the study parameters while the study was under way and thereafter (see Table 1).

**Table 1:** Changes of protocol in the trial registry

	Registration first posted Jan 13, 2012	Last modification in registration data and date	Protocol uploaded Sep 19, 2019	Paper published in October 2020
Participants	600	150 Jul 27, 2016	300	150
Treatment arms	2	3 Jul 27, 2016	3	3
Exclusion criteria*	1	(Unmodified)	9	20
Follow up time for Quality of life (primary endpoint)	2 years	18 weeks Oct 29, 2020 (together with results)	18 weeks	18 weeks
Number of included types of cancer	3	(Unmodified)	1	1

*Each criterion counted separately

The authors did not indicate any substantial modification of their study-parameters in their paper.

The protocol was uploaded to the register on Sep 19, 2019, about two months after data collection was completed where further modifications were introduced (follow-up time for quality of life (QoL) which is the primary endpoint, number of included cancer types). Oddly enough the date given in this document is Jan 10, 2011, a full year before the study was first registered on Jan 13, 2012. This seems misleading, because the final paper does reflect some of the parameters that were disclosed in the protocol while the data used during first registration do not. (Note: yet another version of the protocol was uploaded to the registry on June 14, 2021, but bearing the date of Feb 6, 2014 with all the parameters identical to the published paper. However these modifications were not updated in the registry. Because this protocol was uploaded only long after the final paper was published, we did not include it in this discussion.)

In the published paper, the authors describe 20 exclusion criteria while only one was given in the first registration and none was introduced during any of the seven modifications of the registration data that followed until the final results were uploaded. This raises serious concerns about selection bias.

The inferior survival of the placebo group derives from an unusually high number of deaths within the first nine weeks alone while the treatment groups performed comparably afterwards. This might be the result of some selection bias.

The Consort diagram (Fig. 3 of the original paper) seems incomplete and misleading. Not only compared to Fig. 2 (see below) data seem to be missing. The authors felt inclined to additionally introduce 19 exclusion criteria while the trial was under way but oddly enough none of the 158 patients assessed for eligibility seems to have met any. Only after randomization were eight patients excluded: all due to the same criterion of “sensitizing EGFR mutations or ALK translocations”.

The authors reduced the follow up time of 2 years for QoL to only 18 weeks which raises concerns about incomplete outcome reporting.

Figure 2 of the original publication indicates that there was a group of patients that “refused to participate”, but somehow gave their informed consent to receive verum. Their number is not included in the Consort diagram nor is it given in the text of the paper. In fact, data of these patients would be important to assess the overall effect of the adjunct treatment.

We conclude that the outstanding results of the study may well be due to some serious bias arising from the apparently numerous but undisclosed modifications of the protocol while the study was under way. The authors’ conclusion about the positive influence of the additional homeopathic treatment on the patients’ QoL and survival is unjustified until they are confirmed by an independent and rigorous replication.

## Frass et al. (2015)

We rated this study as high risk of bias in five of eight domains, and the authors discuss that our ratings concerning blinding and allocation concealment are unwarranted. as well as our rating on comparability of treatments and group differences.

The authors remark that “In a cohort study, after randomization, patients are assigned to either treatment or non-treatment group. Therefore, concealing is simply not possible, not necessary and **cannot affect the results**.” (highlighting added). Do we really have to explain why this is false? However, this indicates a deep misunderstanding of essentials of evidence-based medicine by leading researchers of homeopathy.

Comparability of groups: the participants were informed that this is a study on homeopathy. We assume that only patients with at least some positive attitude towards homeopathy enrolled which most probably would result in a selection bias. That a medical university conducts such a trial might have added to the participants' notion. This entails also that those participants randomized in the control arm without any additional attention or care might be disappointed because something possibly helpful was withheld from them, which may lead to some nocebo effect and effect the patient's subjective rating of quality of life and general health which were the endpoints of this study.

The presentation of the outcome according to Table 2 seems not to include data from all patients. “The values reported here are evaluated at a median age of 57, median baseline value of the respective outcome and without chemotherapy, metastases or any other CAM treatment.” Yet, as shown in Table 1 of the manuscript, about half of patients received chemotherapy, a quarter had metastases. Apparently results of a quite important subgroup of patients with either metastases and/or undergoing chemotherapy are not presented.

At first glance, the information on inclusion criteria (adult patients prior to first treatment) is in some contrast to the data in the demographic Table 1: Only patients with stage III and IV cancer were included and 44% (intervention group) and 54% (control arm) had chemotherapy while 13% and 18% resp. had radiotherapy.

The results are not presented in absolute numbers but only as pre-post differences. As data on comparability at baseline are missing, we cannot establish whether differences between intervention and control are due to homeopathic treatment or already existed at baseline (as for example shown in demographic data on age).

Drop out in both groups between first and third homeopathic consultation are high: 29% in intervention (lost between first visit and third visit) and 19% in control group. This is not discussed in the study. It seems possible that larger number of drop outs in the intervention arm may be due to patients disappointed by the lack of improvement by homeopathy. Anyway, the larger number of dropouts should have been analyzed, because with cancer patients in stages III and IV the number of dropouts may well be caused by the deaths or by progression of the disease and thus may indicate severe harm by homeopathy.

At least the difference in dropouts may be indication of some systematic differences between both groups which would forbid multiple imputation of missing data as performed in this study.
